# Efficacy of amisulpride for depressive symptoms in individuals with mental disorders: A systematic review and meta‐analysis

**DOI:** 10.1002/hup.2801

**Published:** 2021-06-03

**Authors:** Caroline Zangani, Barbara Giordano, Hans‐Christian Stein, Stefano Bonora, Armando D'Agostino, Edoardo Giuseppe Ostinelli

**Affiliations:** ^1^ Oxford Health NHS Foundation Trust, Warneford Hospital Oxford UK; ^2^ Department of Psychiatry University of Oxford Oxford UK; ^3^ Oxford Precision Psychiatry Lab, NIHR Oxford Health Biomedical Research Centre Oxford UK; ^4^ Department of Health Sciences University of Milan Milan Italy

**Keywords:** antipsychotics, depression, dysthymia, schizofrenia

## Abstract

**Background:**

Depressive symptoms occur in several psychiatric disorders, often in the absence of a formal diagnosis of depression. We aimed to evaluate the efficacy and the tolerability of amisulpride, both alone and as augmentation therapy, in the treatment of depressive symptoms in individuals with any major psychiatric disorder.

**Methods:**

We searched PubMed, Embase, PsycINFO, GreyLit, OpenGrey and ProQuest up to March 2020 for randomised controlled trials focussing on the treatment of an acute depressive episode in any major psychiatric disorder. A random‐effect meta‐analysis was performed to synthesize the findings on depressive symptoms (primary outcome), response rate and tolerability.

**Results:**

We retrieved 11 studies including 2065 patients with a diagnosis of dysthymia (eight studies), major depression (one study) or schizophrenia (two studies). Amisulpride 50 mg/day was associated with a larger reduction of depressive symptoms compared to placebo (standardised mean difference [SMD] = −0.70, CI 95% −0.92, −0.49; *I* ^2^ = 0.0%), and was found to be comparable to selective serotonin reuptake inhibitors (SSRIs; SMD = −0.08, CI 95% −0.23, 0.06, *I* ^2^ = 0.0%), amineptine, imipramine and amitriptyline in the treatment of dysthymia (three studies, not pooled). In individuals with schizophrenia, amisulpride administered at higher doses (>400 mg/day) was comparable to olanzapine and risperidone (two studies, not pooled). In terms of tolerability, amisulpride was superior to placebo for dysthymia (odds ratio [OR] = 3.94, CI 95% 1.07, 14.48; *I* ^2^ = 0.0) and comparable with SSRIs (OR = 0.94, CI 95% 0.55, 1.62; *I* ^2^ = 0.0%).

**Conclusion:**

Treatment with amisulpride could be a valid choice for selected individuals with dysthymia or depressive symptoms in the context of schizophrenia. More studies on the efficacy and tolerability of amisulpride are needed to draw firm conclusions on its potential benefits in other psychiatric disorders.

## 1 BACKGROUND

The term 'depression' is widely used to describe a clinical spectrum, ranging from subsyndromal isolated depressive symptoms to major depressive disorder (Busch et al., 2013; Vos et al., 2012). It is a major public health problem, considering its high prevalence and severe consequences for individuals and society (Cuijpers & Smit, 2008; Henderson & Pollard, 1992; Judd et al., 1996; Kessler et al., 2011; Murray et al., 2012, 2013). In a recent community cohort study, 54.4% of the sample met lifetime criteria for any DSM‐5 depressive disorder (APA, 2013; Vandeleur et al., 2017). Clinically, depression could present alone or in the context of other diagnoses. Indeed, depressive symptoms and depression co‐occurrence have been reported to be very common in other psychiatric disorders, such as anxiety disorders (Nordahl et al., 2018; Ratnani et al., 2017), post‐traumatic stress disorder (PTSD; Armenta et al., 2019; Campbell et al., 2007), and schizophrenia (SCZ), especially during the first psychotic episode (Häfner et al., 2015).

Although many antidepressant medications are available (Cipriani et al., 2018), a significant proportion of individuals with a depressive episode do not respond to the first treatment (Rush et al., 2006), with up to one‐third eventually classified as having treatment‐resistant depression (Al‐Harbi, 2012). Among the antidepressant drugs, agomelatine has attracted interest due to its efficacy via an alternative mechanism of action (Pompili et al. 2013).

Besides conventional first‐line treatment with antidepressants, second‐generation antipsychotics (SGA), and in particular amisulpride (AMS), have been used in clinical practice, alone or as augmentation, to treat depressive symptoms (Ravindran et al., 2007; Simons et al., 2017). AMS is a substituted benzamide derivative with a higher affinity for dopamine D2/D3 receptors in limbic rather than in nigrostriatal structures, which has been related to the low incidence of extrapyramidal side effects, especially at low doses (Lecrubier, 2004). It shows a double mechanism of action. At low dosages it blocks the D2/D3 autoreceptors enhancing dopamine transmission, while high dosages reduce the transmission by antagonising the postsynaptic receptors (McKeage & Plosker, 2004). For this reason, AMS is considered different to other SGA, such as olanzapine and risperidone, which are pure antagonist, but also to partial agonists, such as aripiprazole. Several authors suggested this double mechanism might explain the beneficial effect of AMS on positive symptoms of SCZ at high doses and on negative and depressive symptoms at low doses (McKeage & Plosker, 2004; Stahl, 2013, 2018). AMS might also be a partial agonist of dopamine 2 receptors (Stahl, 2013, 2018) and, unlike other atypical antipsychotics, does not have potent actions at 5‐HT2A or 5‐HT1A receptors but at 5‐HT2B and 5‐HT7 receptors (Abbas et al., 2009; Stahl, 2013, 2018). Finally, AMS has a renal metabolism, with 25%–50% of the dose eliminated unchanged with urine (Rosenzweig et al., 2002).

Some evidence reported the antidepressant properties of AMS in the treatment of dysthymia, SCZ with co‐occurrence of a depressive episode and depressive symptoms in chronic diseases, such as fibromyalgia and cancer (Calandre & Rico‐Villademoros, 2013; Kim et al., 2007; Montgomery, 2002; Torta et al., 2007). Moreover, AMS is approved for treating dysthymia in Italy and other European countries (Table 1; Pani & Gessa, 2002; Rittmannsberger, 2019).

**TABLE 1:** Availability of AMS in different countries

Country	Availability (psychiatric indication)
Europe	AMS is indicated for the treatment of acute or chronic schizophrenic disorders in the following countries:
	‐ Austria
	‐ Belgium
	‐ Bulgary
	‐ Croatia
	‐ Cyprus
	‐ Czech Republic^a^ (dysthymia)
	‐ Denmark
	‐ Estonia
	‐ France
	‐ Germany
	‐ Greece
	‐ Iceland
	‐ Italy^a^ (dysthymia)
	‐ Latvia
	‐ Lithuania
	‐ Luxembourg
	‐ Norway
	‐ Poland
	‐ Portugal^a^ (dysthymia)
	‐ Romania
	‐ Slovakia
	‐ Slovenia
	‐ Spain
	‐ Switzerland
	‐ United Kingdom
United States^b^	Not available
Canada	Not available
Japan	Available (acute or chronic schizophrenic disorders)
China	Available (acute or chronic schizophrenic disorders)
Russia	Available (acute or chronic schizophrenic disorders)

*Note:* Sources: European Medicines Agency (2020), Food and Drugs Administration (2020), National Centres for Advancing Translational Sciences (2020), drugs.com (2020), Generic Drugs (2018).

Abbreviation: AMS, amisulpride.

^a^ AMS is licensed for dysthymia only in some European countries (e.g., Italy, Czech Republic, Portuga; Rittmansberger, 2019).

^b^ Approved for use in the United States in February 2020 only for treatment and prevention of Postoperative Nausea and Vomiting.

Notwithstanding its use in clinical practice, few high‐quality data on the use of AMS in dysthymia are available (Komossa et al., 2010; Kriston et al., 2014). Furthermore, evidence is still required to establish the efficacy and safety of this molecule across a broader spectrum of diagnoses, including acute depressive episodes (Rittmannsberger, 2019).

The current systematic review and meta‐analysis aimed to assess the efficacy and tolerability profiles of AMS, both as monotherapy and augmentation therapy, in the treatment of acute depressive episodes in individuals with a major mental health disorder.

## 2 METHOD

The systematic review was conducted following the recommendations of the MOOSE and PRISMA statements (see Appendix S1; Moher et al., 2009; Stroup et al., 2000). The protocol is available on PROSPERO with the number CRD42020177918.

### 2.1 Search methods

We searched PubMed, Embase, PsycINFO, GreyLit, OpenGrey and ProQuest from inception until 21st March 2020 for published and unpublished records using relevant keywords and thesauri (see Appendix S2 for the full search strategy). We inspected the reference lists of the records identified from our search to retrieve any additional relevant study.

### 2.2 Selection criteria

#### 2.2.1 Study types

We included only randomised controlled trials (RCTs). No time or language restriction was applied.

#### 2.2.2 Population

We included studies recruiting adult individuals with any primary psychiatric diagnosis (i.e., mood disorders, SCZ spectrum diagnosis, anxiety spectrum diagnosis, obsessive‐compulsive disorder, PTSD), presenting acute depressive episodes/symptoms. Studies comprising individuals with mixed mental health diagnoses (i.e., individuals with different psychiatric diagnoses) were included if at least 80% of the sample had the same diagnosis. Patients with depressive symptoms due to a primary physical condition (e.g., cancer, chronic condition, multiple sclerosis) or with a personality disorder as the solely diagnosis were excluded.

#### 2.2.3 Intervention

AMS, administered alone or as augmentation of the usual treatment. Augmentation studies were only considered if usual treatment was stable prior to randomisation and balanced between the randomised groups. We considered eligible any dosage within the therapeutic range (25–1200 mg; fixed and flexible dosages) and any route of administration.

#### 2.2.4 Comparison

Placebo or any other drug. We excluded studies comparing AMS with non‐pharmacological interventions unless there was one or more pharmacological comparison.

#### 2.2.5 Outcome

Our primary outcome was the reduction of the acute depressive symptomatology assessed by validated scales, as a measure of the efficacy.

Our secondary outcomes were:

‐ Response rate, as defined by the original authors
‐ Tolerability, defined as the number of dropout due to an adverse effect

### 2.3 Selection of studies, data extraction and assessment of study quality

At least two authors (BG, CZ, HCS, SB) independently performed both the abstract screening and the full‐text screening phases. Any disagreement was resolved by consensus or by consultation with another member of the review team (ADA, EGO).

At least two team members (BG, CZ, HCS, SB) independently extracted data and study characteristics according to a pre‐planned data extraction form. Any difference in the extracted data was discussed and resolved by consensus. Articles referring to the same trial were merged to avoid double‐counting.

We attempted to contact the original authors where further information or data were missing and deemed potentially relevant (Appendix S3).

We assessed the quality of the included studies using the Risk Of Bias 2 (ROB2) tool (Sterne et al., 2019).

### 2.4 Statistical analysis

For continuous data, we performed a random‐effects meta‐analysis of the endpoint or change mean depressive symptoms scores. We extracted data for both endpoint and change scores, prioritising the first when both were available. Should different scales be employed, we aim at providing the quantitative synthesis employing the Hedge's *g* standardised effect size. Dichotomous data were pooled using a random‐effects meta‐analysis of the event rate of interest.

Consistency between studies was measured with *I* ^2^ statistics, following the Cochrane Handbook thresholds for the interpretation (Deeks et al., 2020). All the statistical analyses were performed using Stata (StataCorp, 2015). The full code is available upon request to the contact author.

We evaluated the transdiagnostic potential of AMS following the TRANSD criteria, as it has been recently done for aripiprazole (Solmi et al. 2020).

## 3 RESULTS

**FIGURE 1:**
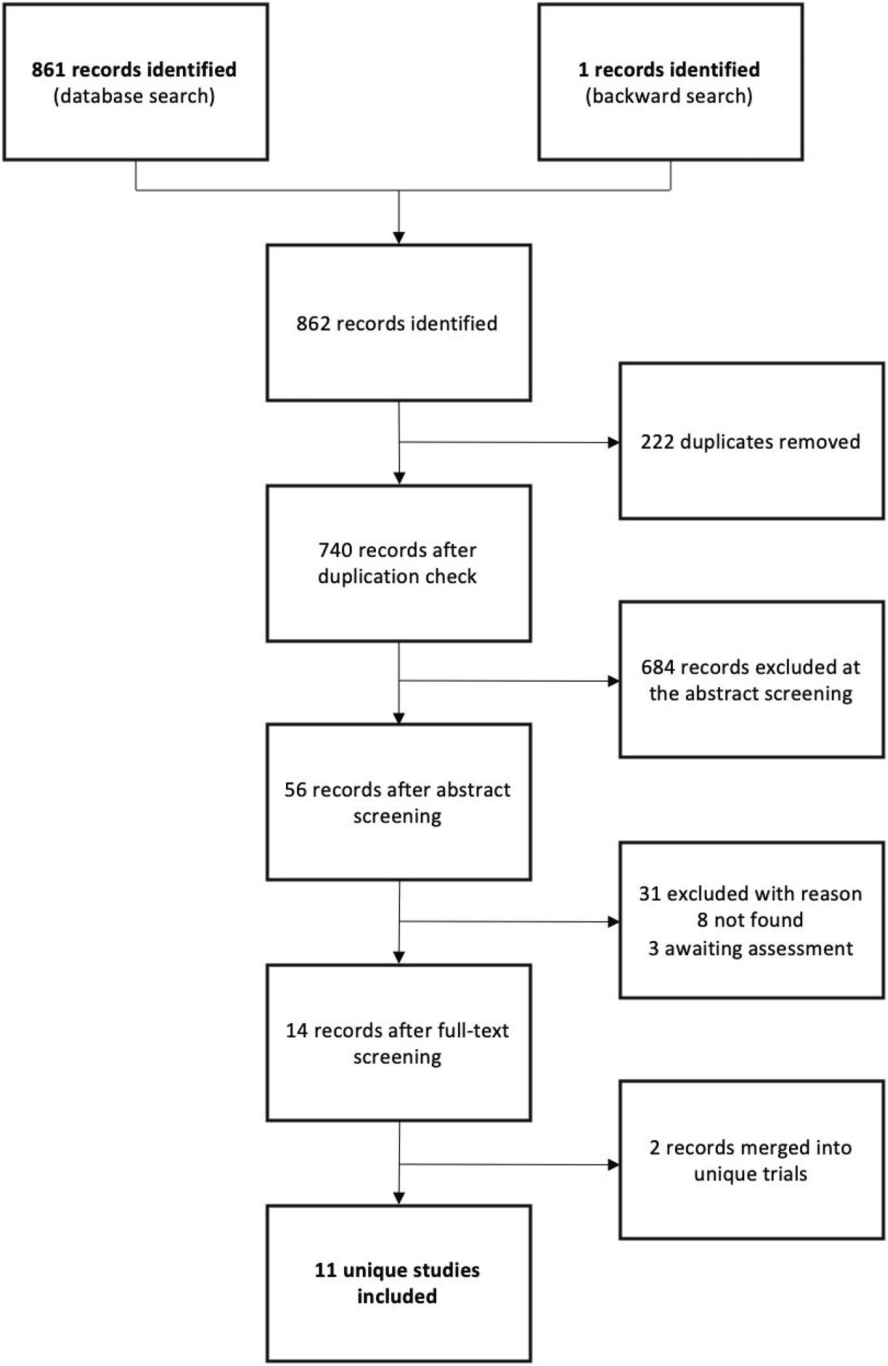
PRISMA flowchart

Our search identified 862 records for the screening (Figure 1). After the duplication check and the screening processes, a total of 57 potentially eligible studies were kept for further examination. Ten full‐text articles could not be retrieved, so 47 papers were examined in full‐text. Of them, 31 were excluded and three remained in ‘awaiting assessment' since no sufficient information for the inclusion could be obtained (Appendix S4). We contacted a total of five authors, but no further data was acquired (Appendix S3). Finally, we included 11 RCTs (dating from 1997 to 2007) with a total of 2065 participants. Of them, eight studies included patients with a diagnosis of dysthymia, one study included patients with major depression disorder (MDD) and two included patients with SCZ. An overview of the characteristics of the included studies is presented in Table 2. Overall, we performed two meta‐analyses on dysthymia (vs. placebo and selective serotonin reuptake inhibitors [SSRIs]), while for all the other diagnostic domains there was either an insufficient number of studies (*k* ≤ 1) or lumping the comparators altogether were considered not justified by available evidence (i.e., olanzapine and risperidone; amineptine, imipramine and amitryptiline). Due to this, TRANS‐D criteria were not applicable.

**TABLE 2:** Studies included in this review (by first author surname)

Author (year)	Drug #1 (mean dose)	Drug #2 (mean dose)	Drug #3 (mean dose)	Type of treatment	Sample size (n)	Diagnosis	RCT design	Duration (weeks)	Rating Scale #1	Rating Scale #2
Amore & Jori (2001)	Amisulpride (50 mg)	Sertraline (75 mg)	‐	Acute, oral	313	Dysthymia	Double‐blind, parallel‐group, multicentre	12	MADRS	HDRS 17‐item
Bellino et al. (1997)	Amisulpride (50 mg)	Sertraline (50 mg)	‐	Acute, oral	49	Dysthymia (elderly subjects)	Open‐label, parallel‐group, single‐centre	24	GDS	HDRS 21‐item
Boyer et al. (1999)	Amisulpride (50 mg)	Amineptine (200 mg)	Placebo (−)	Acute, oral	323	Dysthymia	Double‐blind, parallel‐group, multicentre	12	MADRS	‐
Cassano & Jori (2002)	Amisulpride (50 mg)	Paroxetine (20 mg)	‐	Acute, oral	277	Major depressive disorder	Double‐blind, parallel‐group, multicentre	8	MADRS	HDRS 17‐item
Kim et al. (2007)	Amisulpride (458,3 mg)	Risperidone (4,2 mg)	‐	Acute, oral	87	Schizophrenia	Open‐label, parallel‐group, multicentre	12	CDSS	BDI
Lecrubier et al. (1997)	Amisulpride (50 mg)	Imipramine (100 mg)	Placebo (−)	Acute, oral	219	Dysthymia	Double‐blind, parallel‐group, multicentre	24	MADRS	‐
Ravizza L. (1999)	Amisulpride (50 mg)	Amitriptyline (50 mg)	‐	Acute, oral	253	Dysthymia	Double‐blind, parallel‐group, multicentre	24	MADRS	ERD
Rocca et al. (2002a)	Amisulpride (50 mg)	Paroxetine (20 mg)	‐	Acute, oral	118	Dysthymia	Open‐label, parallel‐group, single‐centre	8	MADRS	HDRS 21‐item
Rocca et al. (2002b)	Amisulpride (50 mg) + paroxetine (20 mg)	Paroxetine (40 mg)	‐	Acute, oral	60	Dysthymia (non‐responders to paroxetine 20 mg/day)	Open‐label, parallel‐group, single‐centre	12	MADRS	HDRS 21‐item
Smeraldi (1998)	Amisulpride (50 mg)	Fluoxetine (20 mg)	‐	Acute, oral	281	Dysthymia	Double‐blind, parallel‐group, multicentre	12	MADRS	ERD
Vanelle &Douki (2006)	Amisulpride (400 mg)	Olanzapine (10 mg)	‐	Acute, oral	85	Schizophrenia	Double‐blind, parallel‐group, multicentre	8	CDSS	‐

Abbreviations: BDI, Beck Depression Inventory; CDSS, Calgary Depression Scale for Schizophrenia; ERD, Retardation Rating Scale for Depression (Echelle de ralentissement dépressif); GDS, Geriatric Depression Scale; HDRS, Hamilton Depression Rating Scale; MADRS, Montgomery–Asberg Depression Rating Scale; RCT, randomised controlled trial.

### 3.1 Dysthymia

A total of eight parallel‐group RCTs (*n* = 1616), five double and three open, explored the use of AMS in adult patients with dysthymic disorder (Amore & Jori, 2001; Bellino et al., 1997; Boyer et al., 1999; Lecrubier et al., 1997; Ravizza, 1999; Rocca et al., 2002a, 2002b; Smeraldi, 1998). One trial enrolled elderly patients. The F/M ratio ranged from 54.8% to 74.9%. Two three‐arm studies compared AMS to placebo and a tricyclic antidepressant (TCA; i.e., amineptine and imipramine, respectively), while one study compared AMS to amitriptyline. Five studies compared AMS to a SSRIs (i.e., sertraline, fluoxetine and paroxetine). In all these trials AMS was studied as monotherapy, except in one study where it was used as augmentation to paroxetine. In all trials, AMS was administered at the fixed dose of 50 mg per day. Five trials were double‐blind and multicentric, while three adopted an open‐label, single‐centre design. The most widely used rating scale for assessing depressive symptoms was the Montgomery–Asberg Depression Rating Scale (MADRS; seven out of eight studies). The other scales were the Hamilton Depression Rating Scale (HAMD) in both the 17‐ and the 21‐item versions, the Retardation Rating Scale for Depression (Echelle de Ralentissement Depressif) and the Geriatric Depression Scale in one study.

#### 3.1.1 AMS versus placebo

Two double‐blind RCTs (*n* = 358) contributed to this outcome (Boyer et al., 1999; Lecrubier et al., 1997). In both studies AMS was administered at the fixed dose of 50 mg/day.

**FIGURE 2:**
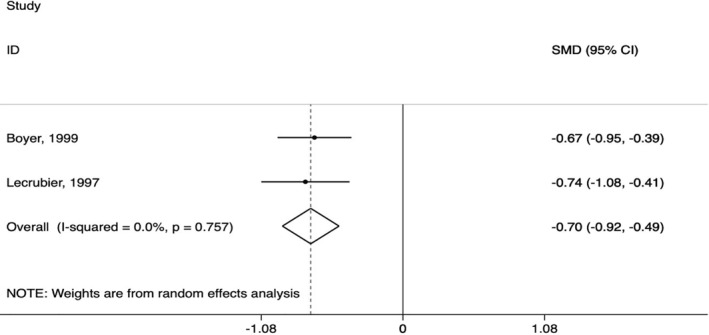
Amisulpride versus placebo in dysthymia (primary outcome)

AMS resulted in lower depressive symptoms compared to placebo in individuals with dysthymia (SMD = −0.70, CI 95% −0.92, −0.49; *I* ^2^ = 0.0%; Figure 2). Both studies were evaluated as at high of bias (Appendix S5).

Regarding our secondary outcomes, individuals allocated to AMS experienced a significantly higher response rate compared to those allocated to placebo (OR = 3.38, CI 95% 2.17, 5.27; *I* ^2^ = 0.0%), as well as a higher risk to dropout due to adverse events (OR = 3.94, CI 95% 1.07, 14.48; *I* ^2^ = 0.0%) (Appendix S6, Figures S1 and S2).

#### 3.1.2 AMS versus SSRIs

We identified five RCTs (*n* = 821) comparing AMS and SSRIs (i.e., fluoxetine, sertraline and paroxetine; Amore & Jori, 2001; Bellino et al., 1997; Rocca et al., 2002a, 2002b; Smeraldi, 1998). All except one compared the drugs as monotherapy. The last study compared the augmentation of AMS on paroxetine to paroxetine alone. Two RCTs were double blind while three were open. In all the considered studies, AMS was administered at the fixed dose of 50 mg/day.

**FIGURE 3:**
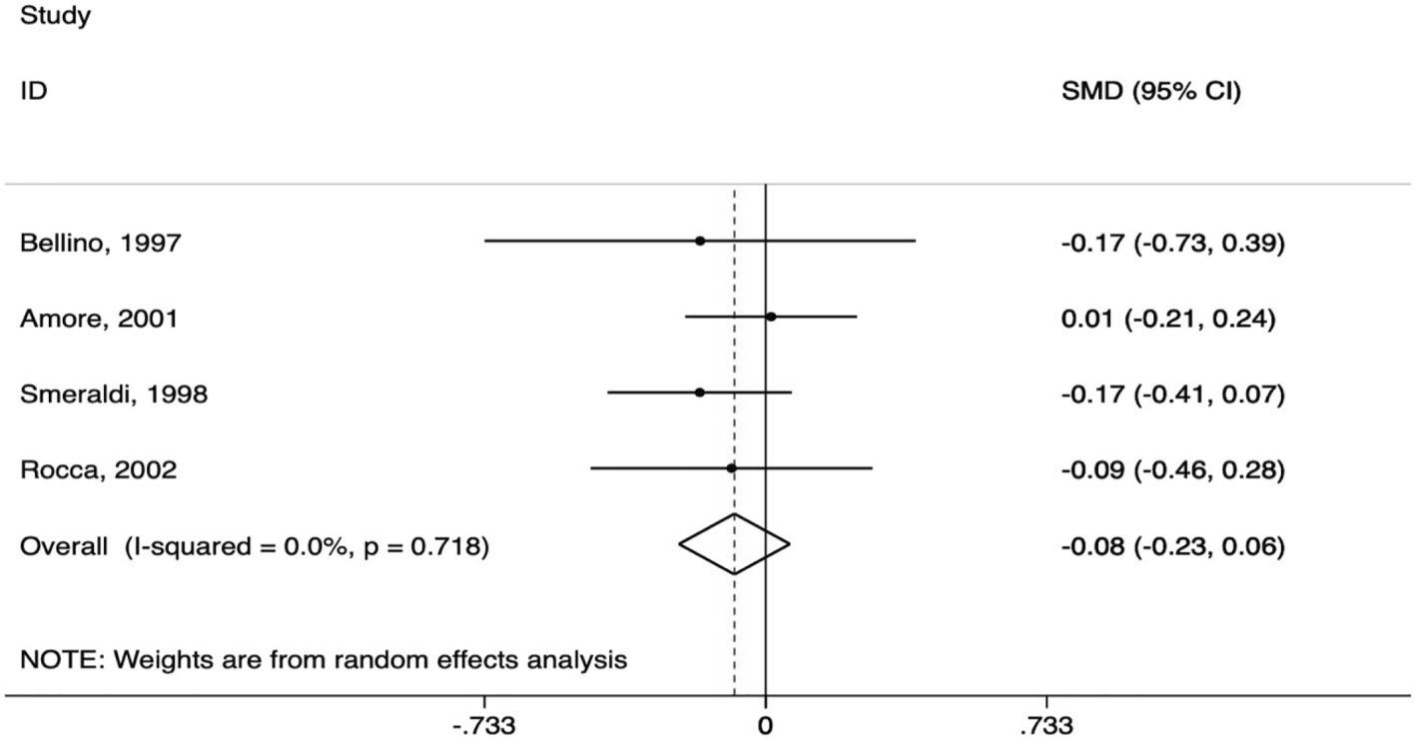
Amisulpride versus selective serotonin reuptake inhibitor in dysthymia (primary outcome)

As shown in Figure 3, no significant difference was found between AMS and SSRIs in terms of reduction of depressive symptoms (SMD = −0.08, CI 95% −0.23, 0.06; *I* ^2^ = 0.0%) in the four studies evaluated AMS as a monotherapy. All studies were ranked as ‘some concerns' at the RoB2 (Appendix S5). The pooled response rate was consistent in not showing a difference between the compared interventions, although considerable levels of inconsistency hinder an accurate interpretation of this effect size (OR = 0.70, CI 95% 0.16,2. 97; *I* ^2^ = 94.3%) (Appendix S6). Finally, when comparing AMS and SSRI in terms of dropout due to adverse events, we could find no significant evidence of difference (OR = 0.94, CI 95% 0.55, 1.62; *I* ^2^ = 0.0%) (Appendix S6).

The only study evaluating AMS as augmentation to paroxetine compared to paroxetine alone found a difference in terms of mean change between treatments no statistically significant (*p* = 0.6149 for the HAMD, *p* = 0.3375 for the MADRS). The percentages of responders were 54% with paroxetine and 56% in the combined treatment group (*p* = 0.9585), while two percentage per group withdrew because of an adverse event.

#### 3.1.3 AMS versus TCAs

Three multicentric, double‐blind, parallel‐group trials (*n* = 614) compared AMS to TCAs in dysthymic disorder (Boyer et al., 1999; Lecrubier et al., 1997; Ravizza, 1999). The included studies employed AMS at either a 50 mg fixed dose (two studies) or flexible dosage (mean 50 mg per day; one study).

Overall, the efficacy of AMS was found comparable to amineptine (MADRS mean change scores −8.6 and −8.2, respectively), imipramine (MADRS mean end scores 12.9 and 14.2, respectively) and amitriptyline (MADRS mean end scores 10.2 ± 8.3 and 10.1 ± 8.5, respectively). All three studies were rated at high risk of bias (Appendix S5). Also, response rate differences were not significant in none of the studies (63.4% vs. 64.5%, 76.5% vs. 68.6% and 60% vs. 62.4% respectively). Tolerability was of 3.9% versus 6.7%, 11% versus 23.3%, and 13.9% versus 12.6%, respectively.

### 3.2 AMS and MDD

Only one study examined the efficacy of AMS in MDD (Cassano & Jori, 2002). This was an 8‐week multicentric, double‐blind, parallel‐group RCT comparing monotherapies of AMS 50 mg per day to paroxetine 20 mg per day in 277 adult outpatients with MDD (mean age 51.2 years, F/M ratio 72.6%). This study found no significant differences between the two drugs either in the reduction in HAMD, MADRS and CGI scores at endpoint (*p* = 0.37, *p* = 0.56 and *p* = 0.51 respectively), or in the response rate, defined as the reduction of at least 50% in the HAMD score (AMS 76% vs. paroxetine 84%, *p* = 0.13). The reported dropout rates were of 5/138 and 6/139 participants per group, respectively. This study was evaluated as ‘some concerns' at RoB2 (Appendix S5).

### 3.3 AMS and SCZ

The efficacy of AMS in reducing acute depressive symptoms in patients with SCZ was examined in two studies (*n* = 172; Kim et al., 2007; Vanelle & Douki, 2006). One study compared AMS 400 mg to olanzapine 10 mg in an 8‐week, multicentric, double‐blind, parallel‐group trial conducted on 85 patients (mean age 34.4 years, F/M 36.5%). In this study, AMS and olanzapine proved comparable efficacy in the reduction of Calgary Depression Scale for Schizophrenia (CDSS) scores (*p* = 0.20). This RCT was rated as ‘some concerns' at the RoB2 evaluation (Appendix S5). Only two patients withdrew due to adverse events in the AMS group, zero in the olanzapine group. The other study compared AMS to risperidone, both given at flexible dosage (mean dosage of 458.3 and 4.2 mg per day, respectively), in a 12‐week multicentric open‐label trial on 87 patients (mean age 35.6 years, F/M 44.8%). In comparison to those on risperidone, patients receiving AMS showed a significantly greater improvement in depressive symptoms (CDSS *p* = 0.027, Beck Depression Inventory *p* = 0.037). The risk of bias was high (Appendix S5). Response rates were also superior in the AMS group (*p* = 0.008). No patient withdrew from the trial due to adverse events in either group.

## 4 DISCUSSION

In the present review, we assessed available studies of AMS for depressive episodes across several mental health conditions. Overall, available evidence suggests AMS might potentially be effective and tolerable as a treatment alternative for individuals with depressive symptoms and an underlying diagnosis of dysthymia, MDD and SCZ. Although depressive features are frequently co‐morbid with other diagnoses (e.g., anxiety disorder, obsessive‐compulsive disorder), AMS was evaluated only for a restricted number of mental health disorders. Hence, a systematic evaluation of the transdiagnostic potential of AMS across and beyond diagnoses using the TRANS‐D criteria (Solmi et al., 2020) could not be assessed due to a limited number of studies.

Most of the included studies focussed on individuals with a dysthymic disorder. The efficacy and tolerability profiles of AMS was overall comparable to both SSRI and TCA antidepressants. The only study evaluating AMS as augmentation to paroxetine compared to paroxetine alone found no difference in terms of response and remission rates, although the group receiving the combined intervention had a significantly greater psychosocial improvement (Rocca et al., 2002b). Despite these findings, its use in the clinical practice is limited. Rittmannsberger (2019) suggested that AMS not being licensed for the treatment of dysthymia in the majority of the Western countries could have contributed its relatively low use. Leveraging the available—albeit limited—evidence, our findings together with peculiar pharmacodynamic properties (e.g., tolerability profile and renal excretion) may support the use of AMS for selected individuals with dysthymia, for instance with significant physical and hepatic comorbidities. More studies are needed to draw a firm conclusion on the clinical role of AMS in dysthymia.

Only one RCT, rated as ‘some concerns' at RoB2, on the treatment of Major Depression was retrieved. It showed that AMS could be a valid alternative for the treatment of MDD (Cassano & Jori, 2002). Indeed, several authors suggested how MDD and dysthymia may lie on the same continuum, with some evidence that the two may intertwine through the clinical history of some patients (Angst et al., 2000; Horwath et al., 1992; Kovacs et al., 1994). Hence, treatment efficacy might be comparable. In a study comparing olanzapine and AMS as augmentation of SSRIs (fluoxetine and sertraline, respectively) for individuals with recurrent depressive disorder, both groups showed a significant reduction of depressive symptoms since Day 10 of the treatment till the end of the study (Day 40) (D'yakonov & Lobanova, 2014). However, the authors reported a minor increase in adverse events with the AMS augmented group (D'yakonov & Lobanova, 2014). These results are in line with a recent report of AMS as an effective and rapid augmentation agent for the treatment of depression, although the efficacy rate might vary between patients (Rittmannsberger, 2019).

In SCZ, the impact of AMS on depressive symptoms is uncertain. Notably, the administered mean dose of AMS was higher than 400 mg/die in both included studies. It has been suggested that lower doses of AMS (<400 mg/die) might be more effective on depressive and negative symptoms, whilst higher dosages for positive symptoms (McKeage & Plosker, 2004). This apparent discrepancy should be considered in light of the individual response to AMS and the gradual activating‐to‐inhibiting transition linked to the pharmacological properties of the drug (Stahl, 2013). Notwithstanding the dosage of AMS administered in the included studies, olanzapine and risperidone did not perform better. The differences in receptor affinities between AMS, olanzapine and risperidone (see the introduction for a comprehensive discussion on AMS pharmacodynamic) highlight the level of complexity of the multi‐receptor networks underlying depressive symptoms (Leggio et al., 2013).

Our findings on the tolerability in patients with SCZ are consistent with the overall low incidence of extrapyramidal symptoms and limited impact on cognitive function on healthy individuals (Rosenzweig et al., 2002). Commonly reported side effects are weight gain and endocrine dysfunctions due to increase in prolactin levels (e.g., galactorrhoea, libido reduction, amenorrhea; Meister et al., 2016; Stahl, 2013, 2018). Its tolerability profile and the renal excretion make AMS suitable as augmentation in patients with complex multi‐pharmacological regimens, or in individuals with significant physical and hepatic comorbidities (Stahl, 2018).

No RCT investigated the potential use of AMS for depressive symptoms in individuals with other psychiatric disorders (e.g., bipolar disorder, anxiety disorder, obsessive‐compulsive disorder), in contrast with the high prevalence of depressive symptoms and depression co‐occurrence in individuals with mental health problems (Armenta et al., 2019; Häfner et al., 2005; Nordahl et al., 2018; Ratnani et al., 2017). Hence, AMS could be further studied as a treatment over the depressive symptoms spectra, also in light of the role of the dopaminergic system in the pathogenesis of depression (Leggio et al., 2013). The activity of AMS as a partial D2 agonist at low doses and as a full D2 antagonist at higher doses (Stahl, 2018) may represent the rationale for its effectiveness in the treatment of depressive symptoms across diagnoses and spectra (Leggio et al., 2013; Stahl, 2018).

The present review presents some limitations. First, eight potentially eligible articles could not be retrieved for a full text assessment. To overcome this limitation, we contacted the original authors. Two original investigators replied but could not provide us with the full text of the publication.

Second, the included studies had overall a moderate to high risk of bias, especially related to missing outcome data and absence of an available protocol. This may be due to the year of publication and the significant changes in the standard for conducting and reporting a trial over time (Schulz et al., 2010). Indeed, the majority of the included studies were published before the 2000.

## 5 CONCLUSION

In summary, we found that AMS might be an effective and tolerable treatment for depression and depressive symptoms. In particular, its use could be evaluated in selected individuals, such as when prioritising renal excretion over hepatic metabolism. This evidence is stronger for dysthymia, and less conclusive for other depressive disorders and depressive episodes in SCZ. Novel high‐quality studies are needed to assess effectiveness and tolerability of AMS as a transdiagnostic agent for the treatment of depressive symptoms.

## CONFLICT OF INTEREST

Edoardo G. Ostinelli has received research and consultancy fees from Angelini Pharma.

## AUTHOR CONTRIBUTIONS

Caroline Zangani and Edoardo Giuseppe Ostinelli wrote the protocol. Barbara Giordano, Caroline Zangani, Hans‐Christian Stein and Stefano Bonora performed the screening process with the supervision of Armando D'Agostino and Edoardo Giuseppe Ostinelli. Edoardo Giuseppe Ostinelli performed the statistical analyses. Finally, all the authors collaborate in writing and revising the manuscript.

## Supporting information

Supplementry Material 1

Supplementry Material 2

Supplementry Material 3

Supplementry Material 4

Supplementry Material 5

Supplementry Material 6

## Data Availability

All materials regarding the review process are available in the Appendix. The full code for the statistical analyses is available upon request.
