# Supplementary material for: Efficacy of amisulpride for depressive symptoms in individuals with mental disorders: A systematic review and meta‐analysis
Source: Hum Psychopharmacol. 2021 Jun 3;36(6):e2801. doi: 10.1002/hup.2801 (PMC8596405; doi:10.1002/hup.2801)
Supplement: Supplementary file 2 — Supplementry Material 2 [file HUP-36-e2801-s005.docx]

**Appendix 2. Search strategy**

The rationale in choosing the search engine/repositories is as follows: 1) MEDLINE (via PubMED) and Embase have been suggested by Cochrane (MECIR C24 - Carol Lefebvre, Julie Glanville, Simon Briscoe, Anne Littlewood, Chris Marshall, Maria-Inti Metzendorf, Anna Noel-Storr, Tamara Rader, Farhad Shokraneh, James Thomas, L. Susan Wieland; on behalf of the Cochrane Information Retrieval Methods Group); 2) ProQuest is a common source when expanding the search to unpublished literature, posters and dissertations; 3) given that our focus was on beyond depression as a diagnosis and extended to depressive symptoms in diagnoses other than depression, we implemented PsycINFO due to its focus on the field of psychology; 4) to accurately and transparently search for unpublished literature (“grey literature”), we searched both open- and restricted-access databases of abstracts, posters, conference proceedings, dissertations and theses: GreyLit, OpenGrey and ProQuest.

**PUBMED**

(((((amisulpride[Title/Abstract]) OR amisulpride[MeSH Terms]) OR solian[Title/Abstract])) AND ((depress*[Title/Abstract]) OR random*[Title/Abstract])) NOT ((animal[Title/Abstract]) NOT human[Title/Abstract])

**EMBASE**

(‘amisulpride’ OR ‘amisulpride’/exp OR amisulpride:ti,ab,kw OR ‘solian’ OR ‘solian’/exp OR solian:ti,ab,kw,tn) AND (Depress*:ti,ab,kw OR random*:ti,ab,kw) AND [embase]/lim NOT ([embase]/lim AND [medline]/lim)

**PSYCinfo**

(depress* OR random).ti,ab AND (amisulpride OR solian).ti,ab

**GREYLit**

Amisulpride

**OpenGrey**

(depress* OR random*) AND (abstract:amisulpride OR abstract:solian)

**ProQuest Dissertations and Theses**

(ab(depress*) OR ab(random*)) AND (ab(amisulpride) OR ab(solian))
