# Supplementary material for: Efficacy of amisulpride for depressive symptoms in individuals with mental disorders: A systematic review and meta‐analysis
Source: Hum Psychopharmacol. 2021 Jun 3;36(6):e2801. doi: 10.1002/hup.2801 (PMC8596405; doi:10.1002/hup.2801)
Supplement: Supplementary file 3 — Supplementry Material 3 [file HUP-36-e2801-s002.docx]

**Appendix 3.** Contacted authors.

|  | Author | Response | Data obtained |
| --- | --- | --- | --- |
| 1 | Bogetto, 1995 | Yes | none |
| 2 | Bogetto, 1997 | Yes | none |
| 3 | Cantelmi, 1996 | Yes | none |
| 4 | Costa-e-Silva, 1990 | No | - |
| 5 | D'yakonov, 2012 | No | - |

**References**

Bogetto F., Fonzo V., Maina G., Ravizza L. Adjunctive fluoxetine or amisulpride improves schizophrenic negative symptoms European Journal of Psychiatry 1995, 9(2):119-126.

Bogetto F., Barzega G., Bellino S., Maina G., Ravizza L. Drug treatment of dysthymia: A clinical study Rivista di Psichiatria 1997, 32(1):1-5.

Cantelmi T., de Angelis F., Pasini A. Assessment of the efficacy and the tolerability of amisulpride in dysthymic disturbances in elderly Ss. [Italian]. Psichiatria e Psicoterapia Analitica 1996, 15(4):365-370.

Costa-e-Silva J.A. Treatment of dysthymic disorder with low-dose amisulpride. A comparative study of 50 mg/d amisulpride versus placebo. Annales de Psychiatrie 1990, 5(3):242-249.

Dyakonov A.L., Lobanova I.V. The comparative study on the efficacy of the combination of serotonin reuptake inhibitor antidepressants and antipsychotics in the treatment of recurrent depressive disorders. Zhurnal Nevrologii i Psihiatrii imeni S.S. Korsakova 2012, 112(11):61-66.
