# Supplementary material for: Efficacy of amisulpride for depressive symptoms in individuals with mental disorders: A systematic review and meta‐analysis
Source: Hum Psychopharmacol. 2021 Jun 3;36(6):e2801. doi: 10.1002/hup.2801 (PMC8596405; doi:10.1002/hup.2801)
Supplement: Supplementary file 4 — Supplementry Material 4 [file HUP-36-e2801-s006.docx]

**Appendix 4.** Studies not included at full-text screening, with reasons.

| **Study** | **Wrong population** | **Wrong intervention** | **Wrong outcome** | **Wrong study type** |
| --- | --- | --- | --- | --- |
| Assion et al. 2008 | X |  |  |  |
| Admon et al. 2017 |  |  | X |  |
| Bogetto et al. 1995 | Full-text not found | | | |
| Bogetto et al. 1997 | Full-text not found | | | |
| Cantelmi et al. 1996 | Full-text not found | | | |
| Carta et al. 2006 |  |  |  | X |
| Colonna et al. 2000 | X |  |  |  |
| Costa e Silva et al. 1990 | Full-text not found | | | |
| Danion et al. 1999 | X |  |  |  |
| Delcker et al. 1990 | X |  |  |  |
| D'yakonov et al. 2014 |  | X |  |  |
| Freeman et al. 1997 | Awaiting List | | | |
| Galderisi et al. 2013 | X |  |  |  |
| Genç et al. 2007 | X |  |  |  |
| Grigorescu et al. 2010 | Awaiting List | | | |
| Hardoy et al. 2010 |  |  |  | X |
| Hofer et al. 2007 | X |  |  |  |
| Kahn et al. 2018 |  | X |  |  |
| Lecrubier et al. 2006 | X |  |  |  |
| Lee et al. 2012 | X |  |  |  |
| Leon et al. 1993 | Full-text not found | | | |
| Leon et al. 1994 | Full-text not found | | | |
| Lewis et al. 2006 | X |  |  |  |
| Loo et al. 1997 | X |  |  |  |
| Mortimer et al. 2004 | X |  |  |  |
| Muller et al. 2002 | X |  |  |  |
| Muller et al. 2002 | X |  |  |  |
| Olié et al. 2006 | X |  |  |  |
| Pawar et al. 2012 | X |  |  |  |
| Peuskens et al. 2002 |  |  |  | X |
| Philipp et al. 2002 |  | X |  |  |
| Rybakowski et al. 2012 |  | X |  |  |
| Saletu et al. 1994 | X |  |  |  |
| Scarzella et al. 1990 |  |  |  |  |
| Sechter et al. 2002 | X |  |  |  |
| Schimmelman et al. 2005 | X |  |  |  |
| Schmidt-Kraepelin et al. 2013 | X |  |  |  |
| Speller et al. 1997 | X |  |  |  |
| Szafranski et al. 2010 |  | X |  |  |
| Zanardi et al. 2006 |  | X |  |  |

**References**

Admon R, Kaiser RH, Dillon DG, et al. Dopaminergic Enhancement of Striatal Response to Reward in Major Depression. *Am J Psychiatry*. 2017;174(4):378‐386

Assion HJ, Reinbold H, Lemanski S, Basilowski M, Juckel G. Amisulpride augmentation in patients with schizophrenia partially responsive or unresponsive to clozapine. A randomized, double-blind, placebo-controlled trial. *Pharmacopsychiatry*. 2008;41(1):24‐28

Bogetto, F., Fonzo, V., Maina, G., & Ravizza, L. (1995). Adjunctive fluoxetine or amisulpride improves schizophrenic negative symptoms. The European Journal of Psychiatry, 9(2), 119–127

Bogetto, F. & Barzega, G. & Bellino, S. & Maina, G. & Ravizza, L.. (1997). Drug treatment of dysthymia: A clinical study. 32. 1-5

Cantelmi T., De Angelis F., Pasini A. Valutazione dell’efficacia e della tollerabilità dell’amisulpride nei disturbi distimici dell’anziano. Psichiatria e Psicoterapia Analitica 15,4:365-370 1996

Carta MG, Zairo F, Mellino G, Hardoy MC, Vieta E. An open label follow-up study on amisulpride in the add-on treatment of bipolar I patients. *Clin Pract Epidemiol Ment Health*. 2006;2:19. Published 2006 Aug 24

Colonna L, Saleem P, Dondey-Nouvel L, Rein W. Long-term safety and efficacy of amisulpride in subchronic or chronic schizophrenia. Amisulpride Study Group. *Int Clin Psychopharmacol*. 2000;15(1):13‐22

Costa-e-Silva JA: Treatment of dysthymic disorder with low-dose amisulpride. A comparative study of 50mg/d amisulpride versus placebo. Annales de psychiatrie 1990; 5:242-49

Danion JM, Rein W, Fleurot O. Improvement of schizophrenic patients with primary negative symptoms treated with amisulpride. Amisulpride Study Group. *Am J Psychiatry*. 1999;156(4):610‐616.

Delcker A, Schoon ML, Oczkowski B, Gaertner HJ. Amisulpride versus haloperidol in treatment of schizophrenic patients--results of a double-blind study. *Pharmacopsychiatry*. 1990;23(3):125‐130

# D’yakonov, A.L., Lobanova, I.V. Comparative Studies of the Efficacy of Combinations of SSRI Antidepressants and Antipsychotics in the Treatment of Recurrent Depressive Disorder. *Neurosci Behav Physi* 44, 195–199 (2014)

Freeman HL. Amisulpride compared with standard neuroleptics in acute exacerbations of schizophrenia: three efficacy studies. *Int Clin Psychopharmacol*. 1997;12 Suppl 2:S11‐S17. doi:10.1097/00004850-199705002-00004

Galderisi S, Mucci A, Bitter I, et al. Persistent negative symptoms in first episode patients with schizophrenia: results from the European First Episode Schizophrenia Trial. *Eur Neuropsychopharmacol*. 2013;23(3):196‐204

Genç Y, Taner E, Candansayar S. Comparison of clozapine-amisulpride and clozapine-quetiapine combinations for patients with schizophrenia who are partially responsive to clozapine: a single-blind randomized study. *Adv Ther*. 2007;24(1):1‐13

Grigorescu, G. & Baloescu, A. & Vasile, D. & Tudor, C. & Vasiliu, O. & Grigorescu, R.. (2010). Standard antidepressant therapy vs association between amisulpride and antidepressant therapy in atypical depression. European Neuropsychopharmacology 20

Hardoy MC, Carta MG. Strategy to Accelerate or Augment the Antidepressant Response and for An Early Onset of SSRI Activity. Adjunctive Amisulpride to Fluvoxamine in Major Depressive Disorder. *Clin Pract Epidemiol Ment Health*. 2010;6:1‐3. Published 2010 Jan 27

Hofer A, Rettenbacher MA, Edlinger M, et al. Outcomes in schizophrenia outpatients treated with amisulpride or olanzapine. *Pharmacopsychiatry*. 2007;40(1):1‐8

Kahn R; Optimise Study Group. 31.1 Optimising the treatment and management of first-episode schizophrenia: the optimise clinical trial. *Schizophr Bull*. 2018;44(Suppl 1):S50.

Lecrubier Y, Quintin P, Bouhassira M, Perrin E, Lancrenon S. The treatment of negative symptoms and deficit states of chronic schizophrenia: olanzapine compared to amisulpride and placebo in a 6-month double-blind controlled clinical trial. *Acta Psychiatr Scand*. 2006;114(5):319‐327

Lee SJ, Lee JH, Jung SW, Koo BH, Choi TY, Lee KH. A 6-week, randomized, multicentre, open-label study comparing efficacy and tolerability of amisulpride at a starting dose of 400 mg/day versus 800 mg/day in patients with acute exacerbations of schizophrenia. *Clin Drug Investig*. 2012;32(11):735‐745

León, C., & León, A. (1993). The clinical profile of dysthymia in a group of Latin American women. *European Psychiatry,* *8*(5), 257-265

León CA, Vigoya J, Conde S, Campo G, Castrillón E, León A. Comparación del efecto de la amisulprida y la viloxacina en el tratamiento de la distimia [Comparison of the effect of amisulpride and viloxazine in the treatment of dysthymia]. *Acta Psiquiatr Psicol Am Lat*. 1994;40(1):41‐49

Lewis SW, Barnes TR, Davies L, et al. Randomized controlled trial of effect of prescription of clozapine versus other second-generation antipsychotic drugs in resistant schizophrenia. *Schizophr Bull*. 2006;32(4):715‐723

Loo H, Poirier-Littre MF, Theron M, Rein W, Fleurot O. Amisulpride versus placebo in the medium-term treatment of the negative symptoms of schizophrenia. *Br J Psychiatry*. 1997;170:18‐22

Mortimer A, Martin S, Lôo H, Peuskens J; SOLIANOL Sudy Group. A double-blind, randomized comparative trial of amisulpride versus olanzapine for 6 months in the treatment of schizophrenia. *Int Clin Psychopharmacol*. 2004;19(2):63‐69

Müller MJ, Wetzel H, Eich FX, et al. Dose-related effects of amisulpride on five dimensions of psychopathology in patients with acute exacerbation of schizophrenia. *J Clin Psychopharmacol*. 2002;22(6):554‐560

Müller MJ, Wetzel H, Benkert O. Differential effects of high-dose amisulpride versus flupentixol on latent dimensions of depressive and negative symptomatology in acute schizophrenia: an evaluation using confirmatory factor analysis. *Int Clin Psychopharmacol*. 2002;17(5):249‐261

Olié JP, Spina E, Murray S, Yang R. Ziprasidone and amisulpride effectively treat negative symptoms of schizophrenia: results of a 12-week, double-blind study. *Int Clin Psychopharmacol*. 2006;21(3):143‐151

Pawar, Ganesh & Phadnis, P & Paliwal, Abhay. (2012). Evaluation of Efficacy, Safety, and Cognitive Profile of Amisulpride Per Se and Its Comparison with Olanzapine in Newly Diagnosed Schizophrenic Patients in an 8-Week, Double-Blind, Single-Centre, Prospective Clinical Trial. ISRN psychiatry. 2012. 703751

Peuskens J, Möller HJ, Puech A. Amisulpride improves depressive symptoms in acute exacerbations of schizophrenia: comparison with haloperidol and risperidone. *Eur Neuropsychopharmacol*. 2002;12(4):305‐310

Philipp M, Lesch OM, Schmauss M, Dose M, Glaser T. Vergleichbare Wirksamkeit von Flupentixol und Risperidon auf schizophrene Negativsymptomatik [Comparative effectiveness of flupenthixol and risperidone on negative symptoms of schizophrenia]. *Psychiatr Prax*. 2003;30 Suppl 2:S94‐S96

Rybakowski JK, Vansteelandt K, Szafranski T, et al. Treatment of depression in first episode of schizophrenia: results from EUFEST. *Eur Neuropsychopharmacol*. 2012;22(12):875‐882

Saletu B, Küfferle B, Grünberger J, Földes P, Topitz A, Anderer P. Clinical, EEG mapping and psychometric studies in negative schizophrenia: comparative trials with amisulpride and fluphenazine. *Neuropsychobiology*. 1994;29(3):125‐135

Scarzella R, Scarzella L, Rovera G G (1990) Amisulpride versus sulpiride: studio clinico in doppio cieco su 68 pazienti a¡etti da disturbo distimico. Giornale di Neuropsicofarmacologia 12: 73-78

Schimmelmann BG, Moritz S, Karow A, et al. Correlates of subjective well-being in schizophrenic patients treated with atypical antipsychotics. *Int J Psychiatry Clin Pract*. 2005;9(2):94‐98

Schmidt-Kraepelin, C., Feyerabend, S., Engelke, C. *et al.* A randomized double-blind controlled trial to assess the benefits of amisulpride and olanzapine combination treatment versus each monotherapy in acutely ill schizophrenia patients (COMBINE): methods and design. *Eur Arch Psychiatry Clin Neurosci* 270, 83–94 (2020)

Sechter D, Peuskens J, Fleurot O, Rein W, Lecrubier Y; Amisulpride Study Group. Amisulpride vs. risperidone in chronic schizophrenia: results of a 6-month double-blind study [published correction appears in Neuropsychopharmacology. 2003 Mar;28(3):611]. *Neuropsychopharmacology*. 2002;27(6):1071‐1081

Speller JC, Barnes TR, Curson DA, Pantelis C, Alberts JL. One-year, low-dose neuroleptic study of in-patients with chronic schizophrenia characterised by persistent negative symptoms. Amisulpride v. haloperidol. *Br J Psychiatry*. 1997;171:564‐568

Szafrański, Tomasz & Jarema, Marek & Olajossy, Marcin & Rabe-Jabłońs, Jolanta & Rybakowski, Janusz. (2010). Depressive symptoms in the first episode of schizophrenia - analysis of polish results of the eufest study. Schizophrenia Research - 117. 503-503

Zanardi R, Smeraldi E. A double-blind, randomised, controlled clinical trial of acetyl-L-carnitine vs. amisulpride in the treatment of dysthymia. *Eur Neuropsychopharmacol*. 2006;16(4):281‐287
