# Supplementary material for: Efficacy of amisulpride for depressive symptoms in individuals with mental disorders: A systematic review and meta‐analysis
Source: Hum Psychopharmacol. 2021 Jun 3;36(6):e2801. doi: 10.1002/hup.2801 (PMC8596405; doi:10.1002/hup.2801)
Supplement: Supplementary file 5 — Supplementry Material 5 [file HUP-36-e2801-s004.docx]

**Appendix 3.** Risk of Bias tool 2.

|  | Risk of bias arising from the randomization process | Risk of bias due to deviations from the intended interventions | Risk of bias due to missing outcome data | Risk of bias in measurement of the outcome | Risk of bias in selection of the reported result | Final score |  |  |  |
| --- | --- | --- | --- | --- | --- | --- | --- | --- | --- |
| Boyer 1999 | 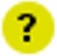 | 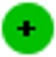 | 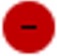 | 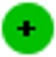 | 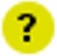 | 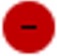 |  | 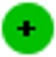 | Low risk |
| Bellino 1997 | 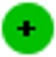 | 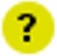 | 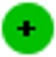 | 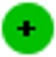 | 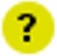 | 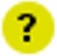 |  | 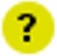 | Some concerns |
| Kim 2007 | 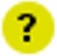 | 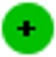 | 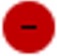 | 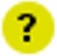 | 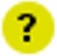 | 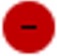 |  | 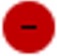 | High risk |
| Vanelle 2006 | 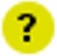 | 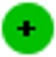 | 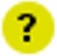 | 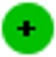 | 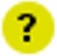 | 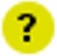 |  |  |  |
| Lecrubier 1997 | 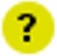 | 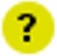 | 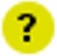 | 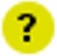 | 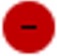 | 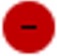 |  |  |  |
| Cassano 2002 | 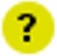 | 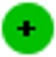 | 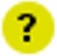 | 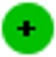 | 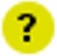 | 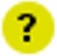 |  |  |  |
| Amore 2001 | 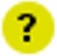 | 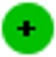 | 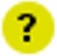 | 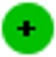 | 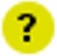 | 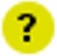 |  |  |  |
| Smeraldi 1998 | 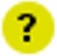 | 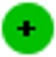 | 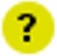 | 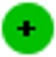 | 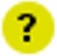 | 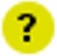 |  |  |  |
| Rocca 2002a | 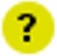 | 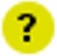 | 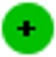 | 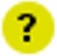 | 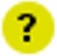 | 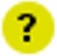 |  |  |  |
| Rocca 2002b | 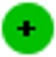 | 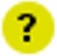 | 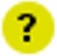 | 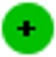 | 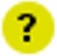 | 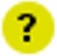 |  |  |  |
| Ravizza 1999 | 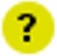 | 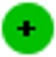 | 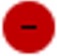 | 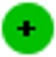 | 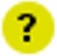 | 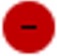 |  |  |  |
