# Supplementary material for: Efficacy of amisulpride for depressive symptoms in individuals with mental disorders: A systematic review and meta‐analysis
Source: Hum Psychopharmacol. 2021 Jun 3;36(6):e2801. doi: 10.1002/hup.2801 (PMC8596405; doi:10.1002/hup.2801)
Supplement: Supplementary file 6 — Supplementry Material 6 [file HUP-36-e2801-s003.docx]

**APPENDIX 6**

**Figure S1 Amisulpride vs Placebo in dysthymia – Response rate**

**
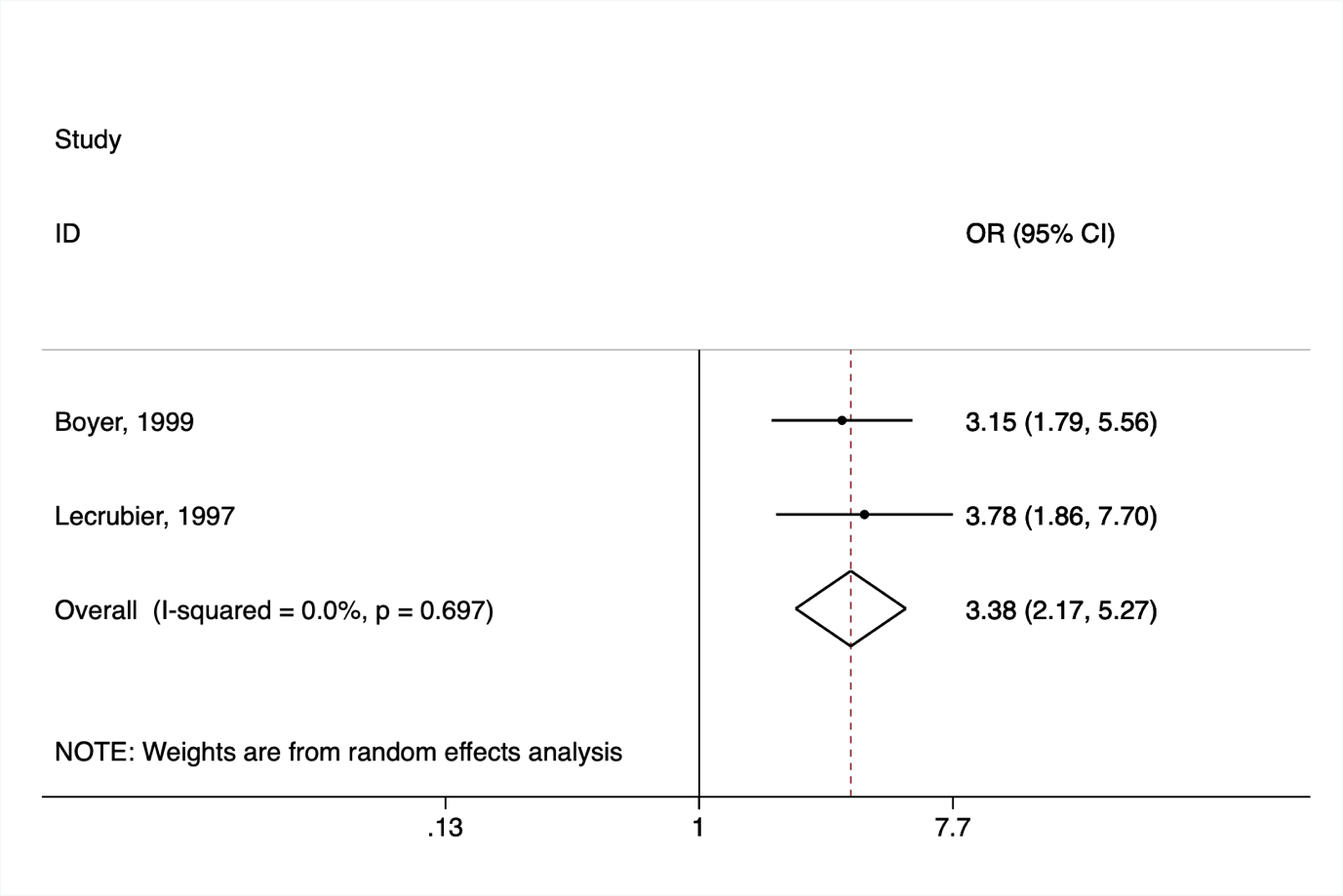
**

**Figure S2 Amisulpride vs Placebo in dysthymia - Dropout rate due to adverse events**

**
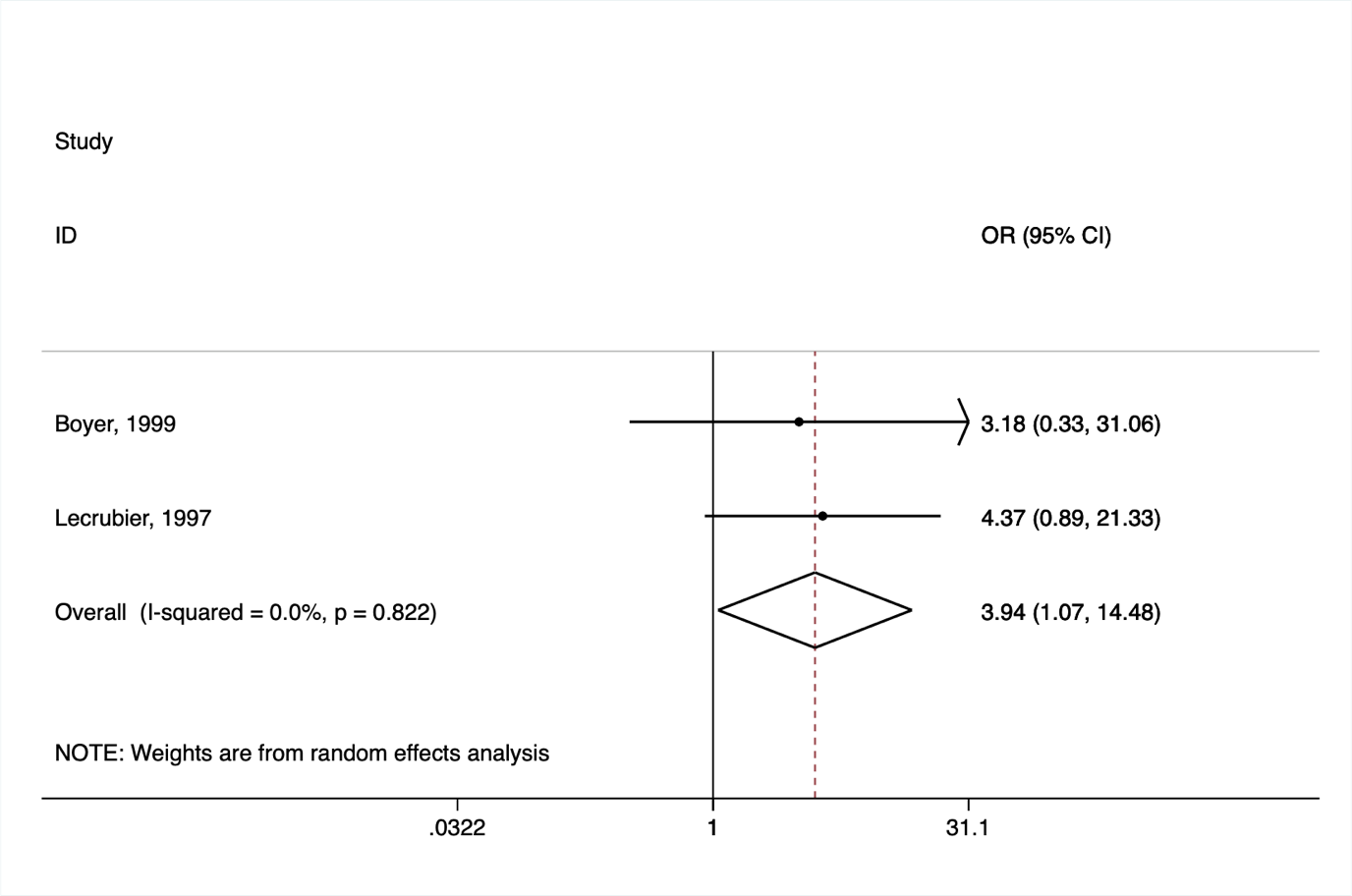
**

**Figure S3 Amisulpride vs SSRI in dysthymia – Response rate**

**
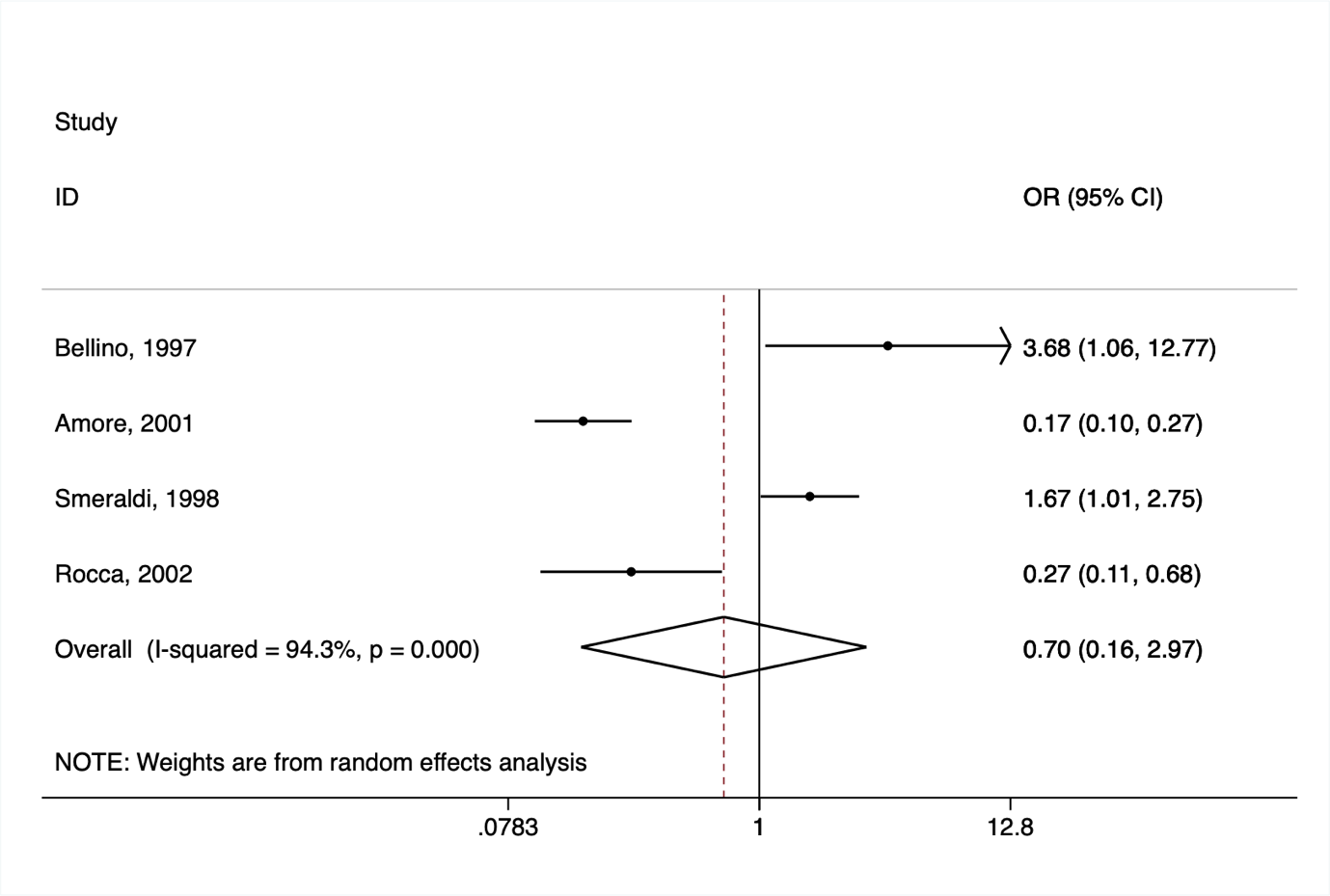
**

**Figure S4 Amisulpride vs SSRI in dysthymia - Dropout rate due to adverse events**

**
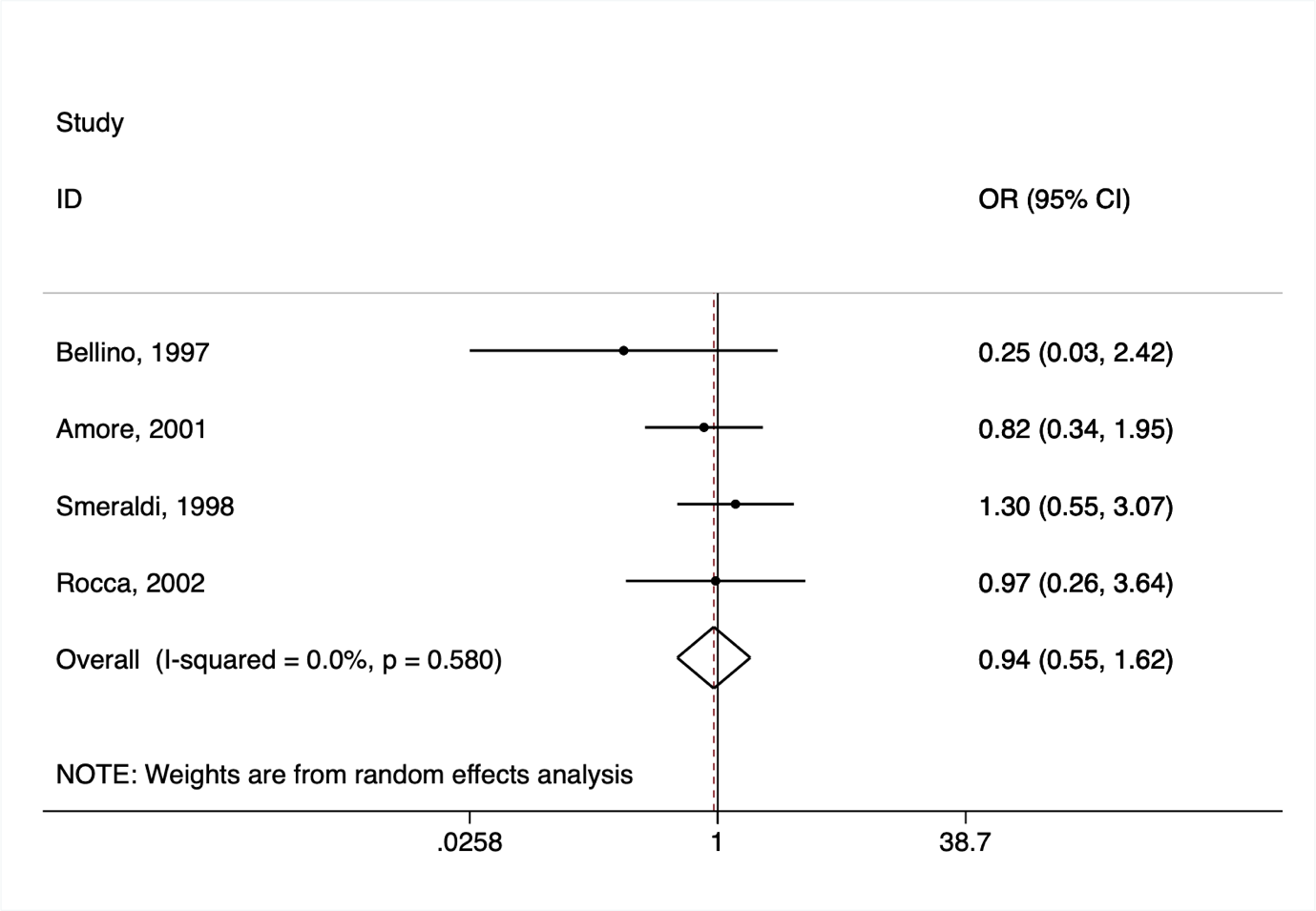
**
